# Human Rights and Covid-19 pandemic

**DOI:** 10.5935/1518-0557.20200041

**Published:** 2020

**Authors:** Carlos Valerio

**Affiliations:** 1 Member of the Latin American Association of Medical Law and of the Association of Medical Law of Costa Rica; 2 Member of the Ombudsman Office of Costa Rica

It should be noted, to begin with, that International Human Rights Law guarantees the right to the highest possible level of healthcare for everyone and, consequently, States are required to take the necessary administrative, legislative, or judicial measures to achieve that goal. Particularly, States must commit to be guarantors of Public Health and provide health care to all in the face of the characteristics of this pandemic (International Covenant on Economic, Social and Cultural Rights, 1966).

International Instruments of Human Rights also recognize that, in the context of serious threats to public health and public emergencies that place life at risk, restrictions on some rights may be justified only when they meet the following requirements: based on legal grounds, strictly necessary, based on scientific evidence, not arbitrary nor discriminatory, of limited duration, respectful of human dignity, subject to revision, and proportionate to achieve their objectives (Organization of American States, 2020). The scale and severity of the Covid-19 pandemic clearly rises to the level of a public health threat that could justify restrictions on certain rights, such as those resulting from the imposition of quarantine or isolation that limit freedom of movement. At the same time, careful attention of the so called “hard core of human rights” is necessary to achieve coherence, transparency and respect for human dignity (Spanish Bioethics Committee, 2020; Office of the United Nations High Commissioner for Human Rights, 2020).

Both the American Convention on Human Rights in its Article 27, as well as the Covenant on Civil and Political Rights in its Article 4, authorize States to implement special measures of exception to satisfy some rights and in turn define those rights that cannot be revoked.

Article 4 of the International Covenant on Civil and Political Rights:

*“1 . In time of public emergency which threatens the life of the nation and the existence of which is officially proclaimed, the States Parties to the present Covenant may take measures derogating from their obligations under the present Covenant to the extent strictly required by the exigencies of the situation, provided that such measures are not inconsistent with their other obligations under international law and do not involve discrimination solely on the ground of race, colour, sex, language, religion or social origin.*

*2. No derogation from articles 6, 7, 8 (paragraphs I and 2), 11, 15, 16 and 18 may be made under this provision.*

*3. Any State Party to the present Covenant availing itself of the right of derogation shall immediately inform the other States Parties to the present Covenant, through the intermediary of the Secretary-General of the United Nations, of the provisions from which it has derogated and of the reasons by which it was actuated. A further communication shall be made, through the same intermediary, on the date on which it terminates such derogation.”*

Article 27 of the American Convention on Human Rights. Suspension of Guarantees:

*“1. In time of war, public danger, or other emergency that threatens the independence or security of a State Party, it may take measures derogating from its obligations under the present Convention to the extent and for the period of time strictly required by the exigencies of the situation, provided that such measures are not inconsistent with its other obligations under international law and do not involve discrimination on the ground of race, color, sex, language, religion, or social origin.*

*2.The foregoing provision does not authorize any suspension of the following articles: Article 3 (Right to Juridical Personality), Article 4 (Right to Life), Article 5 (Right to Humane Treatment), Article 6 (Freedom from Slavery), Article 9 (Freedom from Ex Post Facto Laws), Article 12 (Freedom of Conscience and Religion), Article 17 (Rights of the Family), Article 18 (Right to a Name), Article 19 (Rights of the Child), Article 20 (Right to Nationality), and Article 23 (Right to Participate in Government), or of the judicial guarantees essential for the protection of such rights.*

*3. Any State Party availing itself of the right of suspension shall immediately inform the other States Parties, through the Secretary General of the Organization of American States, of the provisions the application of which it has suspended, the reasons that gave rise to the suspension, and the date set for the termination of such suspension.”*

The mechanism established in Article 27 of the Convention has been applied mainly in Latin American countries for the restoration of Democracy. However, an analogy can be made in the face of a pandemic to apply to the limitation of some rights. Contrary sensu and as the Inter-American Court has indicated, the rights that have not been enumerated in the above articles can be subject to a review in order to determine that there is no abuse of power for their limitation and that they are according to international standards.

The Inter-American Court of Human Rights, in its constant jurisprudence regarding the right to life, has indicated that:

*“One of the obligations that the State must inescapably assume in its position as guarantor, with the aim of protecting and guaranteeing the right to life, is to generate the minimum living conditions compatible with the dignity of the human person and not to produce conditions that hinder or prevent it. In this sense, the State has the duty to adopt positive, concrete measures aimed at satisfying the right to a decent life, especially when it comes to people in situations of vulnerability and risk, whose attention becomes a priority” (Inter-American Court of Human Rights, 2005)*

However, it is worth noting that the activation of the state of exception, as it is known, has rules, for example, that it must be for a limited time, justified, and it must be communicated to the General Secretariat of the Organization of States American for approval. The reason is that the limitation of rights is an extremely sensitive issue for the rights of individuals and democracy.

Furthermore, the General Comment No. 31 [80] of the Covenant on Civil and Political Rights of May 2004 provides that:

*“States Parties must refrain from violation of the rights recognized by the Covenant, and any restrictions on any of those rights must be permissible under the relevant provisions of the Covenant. Where such restrictions are made, States must demonstrate their necessity and only take such measures as are proportionate to the pursuance of legitimate aims in order to ensure continuous and effective protection of Covenant rights. In no case may the restrictions be applied or invoked in a manner that would impair the essence of a Covenant right.”*

The 1984 Syracuse Principles* (American Association for the International Commission of Jurists, 1984), and the General Comments of the United Nations Human Rights Committee on States of Emergency and Freedom of Movement, in particular General Comment No. 29** (Office of the United Nations High Commissioner for Human Rights, 2001), also provide authoritative guidance for restrictions on human rights for reasons of public health or national emergency. In this sense, any measure taken to protect the population that limits people’s rights and freedoms must be legal, necessary, and proportionate. Furthermore, states of emergency must be of limited duration and any reduction in rights must take into account the disproportionate impact on specific populations or vulnerable groups (Human Rights Watch, 2020).

In this regard, the Syracuse Principles specifically state that the restrictions should, at a minimum, be:

- Planned and carried out in accordance with the law
- Directed towards a legitimate objective of general interest
- Be strictly necessary in a democratic society to achieve the objective; the least intrusive and restrictive available to achieve the goal
- Based on scientific evidence and neither arbitrary nor discriminatory in its application, and
- Of limited duration, respectful of human dignity and subject to review.

In this sense, International Human Rights Law, in particular the International Covenant on Civil and Political Rights, requires that restrictions on rights for reasons of public health or national emergency are legal, necessary, and proportionate. Restrictions such as mandatory quarantine or isolation of symptomatic persons must, at a minimum, be carried out in accordance with the law. They must be strictly necessary to achieve a legitimate objective, based on scientific evidence, proportionate to achieve that objective, neither arbitrary nor discriminatory in their application, of limited duration, respectful of human dignity and subject to revision.

Furthermore, the International Health Regulations (IHR 2005), which is the international legal instrument designed to support the protection of all States against the international spread of disease and currently legally binding on 194 States Parties worldwide, provides in Article 2:

*“Article 2 Purpose and scope. The purpose and scope of these Regulations are to prevent, protect against, control and provide a public health response to the international spread of disease in ways that are commensurate with and restricted to public health risks, and which avoid unnecessary interference with international traffic and trade.”*

Regarding the Principles guiding this norm, it is established that its application will fully respect dignity, human rights and fundamental freedoms of people.

## CONCLUSIONS

1. In accordance with International Human Rights Law, both globally and in the Americas, States may issue special measures that restrict human rights.
2. These restrictions are not derogations, they are suspension of guarantees.
3. The imposition of these measures is justified when there is a serious threat to life and health.
4. These restrictions cannot affect the essential core of human rights, that is, the right to life and health.
5. Exceptional measures taken by the State due to the national emergency must be legal, necessary and proportionate. Also strictly necessary to achieve a legitimate objective, based on scientific evidence, and in accordance with international guidelines. Neither arbitrary nor discriminatory in its application, of limited duration and respectful of human dignity.

## Footnotes

* The Syracuse principles are not a treaty, and therefore do not create obligations. They have not been adopted in any United Nations document, as the Paris Principles have been, for example. Its origin is academic. Therefore, the Syracuse principles, in themselves, do not create obligations and States are not obliged to invoke them. They can be considered voluntarily.

** The General Observations, in themselves, do not generate obligations. They are authoritative interpretations of the obligations assumed in each of the human rights instruments ratified by the States and therefore the States are not obliged to report actions vis-a-vis those recommendations or general observations.
